# Oleoresin of Male Fern: Increasing Its Efficacy against Tapeworm

**Published:** 1882-10

**Authors:** 


### OLEORESIN OF MALE FERN: INCREASING ITS EFFICACY AGAINST TAPEWORM.

---

According to E. Dieterich, the frequent failure of oleoresin of male fern as a remedy against tapeworm is to be ascribed to its irrational administration. It has become known that the popular "worm doctors," who use almost exclusively the oleoresin of male fern, and who hardly ever meet with a failure, administer the remedy in conjunction with castor oil, instead of following it by the oil after one or two hours, as is usually done by practitioners. The object is to bring the extract, in an unaltered or undigested condition, in contact with the worm. The experiments which have been made by mixing one part of the oleoresin with two parts of castor oil have been very successful and this mode of administration deserves therefore the preference. Oleoresin of male fern is apt to derange the stomach, and when enveloped partly in the oil is likely to pass it more rapidly. which constitutes another advantage. The mixture has, it is true, an unpleasant taste. This may, however, be disguised by filling it in capsules of about three grams (forty-five grains) each. The dose may be regulated from six capsules (equal to six grams or ninety grains of the oleoresin and twelve grams of castor oil) to seven or eight more, according to circumstances. It is advisable to empty the bowels on the preceding day by a mild purgative, best by castor oil.—*New Remedies.*

